# Rare case of pediatric trauma with hepatic injury managed using gel-foam embolization: a case report

**DOI:** 10.1093/jscr/rjad522

**Published:** 2023-09-20

**Authors:** Asama Rana, James O’Toole, Zamaan Hooda, John Veltri, Elizabeth Kiselak, Benjamin Rebein

**Affiliations:** Department of Surgery, St. Joseph’s University Medical Center, Paterson, NJ 07503, United States; Department of Surgery, St. Joseph’s University Medical Center, Paterson, NJ 07503, United States; Department of Surgery, St. Joseph’s University Medical Center, Paterson, NJ 07503, United States; Department of Surgery, St. Joseph’s University Medical Center, Paterson, NJ 07503, United States; Department of Surgery, St. Joseph’s University Medical Center, Paterson, NJ 07503, United States; Department of Surgery, St. Joseph’s University Medical Center, Paterson, NJ 07503, United States

## Abstract

Nonoperative management for hepatic injuries requires observation and supportive care in the case of hemodynamically stable patients. If there is active bleeding on presentation, hepatic artery embolization is an option to achieve hemostasis in the acute setting. Although interventional radiology procedures are well documented in adults, there is limited literature regarding these procedures in the pediatric population. In this report, we present a case of a pediatric patient who sustained blunt abdominal trauma, resulting in a grade IV liver injury. Treatment involved fluoroscopically guided right hepatic segmental arterial gel-foam embolization.

## Introduction

The liver is the most injured organ in the pediatric populations [1]. A major sequelae of trauma to the liver is hemorrhage and its management is dependent upon the patient’s hemodynamic stability. Hemodynamically unstable patients require immediate exploratory laparotomy. However, for hemodynamically stable patients, various treatment modalities exist, including supportive care and minimally invasive procedures. Common minimally invasive approaches include arterial embolization of the bleeding vessel [2]. Cases of arterial embolization have been widely reported in adult hepatic trauma, but there are few reported cases of pediatric hepatic trauma managed with the same technique [3]. This report describes a case of a pediatric patient with a traumatic grade IV liver laceration treated with gel-foam (gelatin sponge) embolization.

## Case presentation

An 18-month-old female presented to the emergency department after being dragged underneath a car. She presented with low-normal blood pressure and tachycardia, and the patient had a transient response to initial fluid boluses. As part of the initial evaluation, the focused assessment with sonography for trauma scan was positive for fluid in the hepatorenal and splenorenal regions.

**Figure 1:**
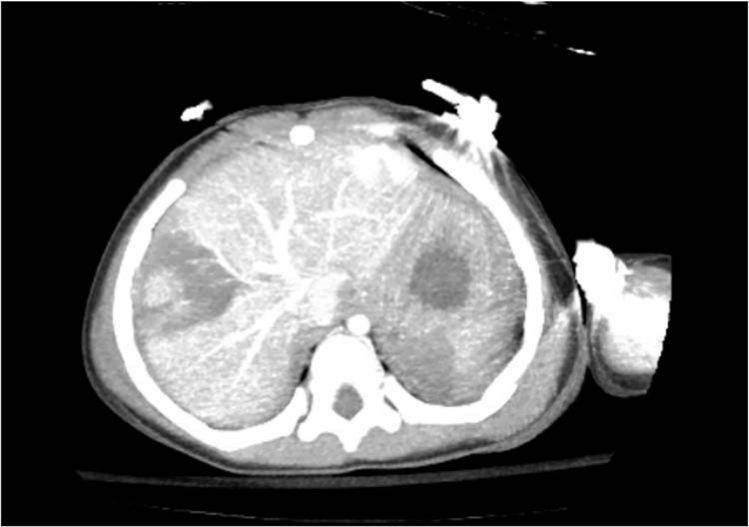
Axial view of CT image revealing liver and splenic injuries.

**Figure 2:**
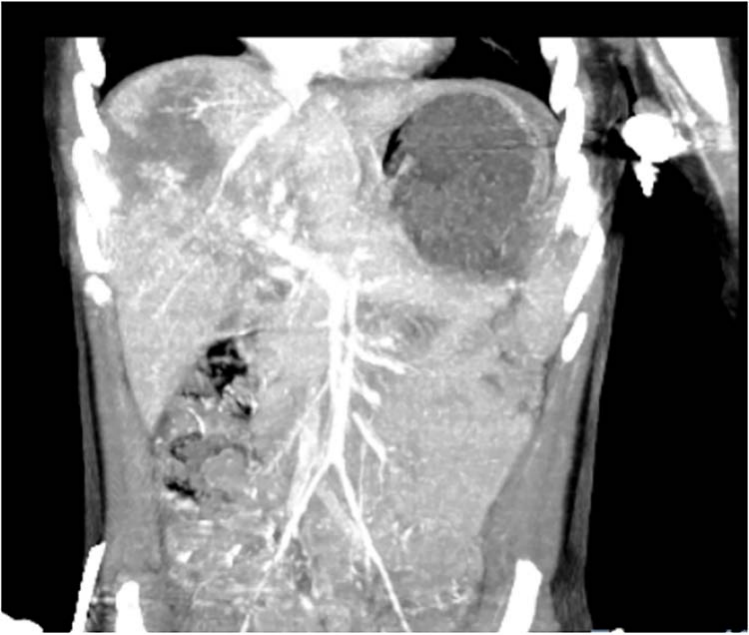
Coronal view of CT image revealing liver and splenic injuries.

**Figure 3:**
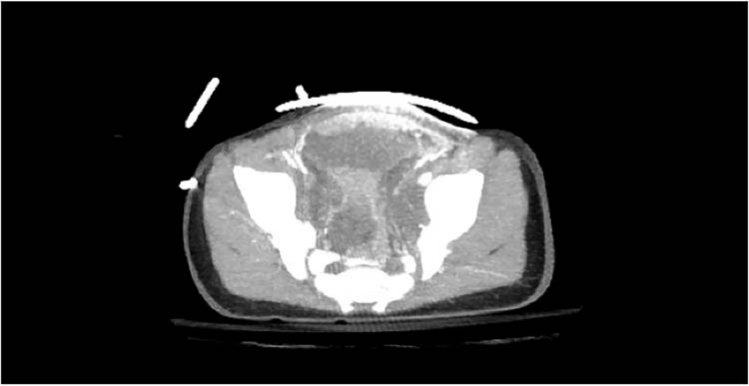
Bladder hematoma adjacent to pubic ramus fracture.

Computed tomography (CT) of the head and spine showed no other injuries. CT chest, abdomen, and pelvis showed a femoral neck fracture, grade IV liver injury, grade II splenic injury, and a fracture of the left iliac wing (Figs 1 and 2), along with a left sided bladder hematoma secondary to a pubic ramus fracture (Fig. 3). Interventional radiology was consulted to evaluate the liver and splenic injuries.

During the procedure, the right common femoral artery was accessed, and the common hepatic artery was selectively cannulated. Diagnostic angiography demonstrated no definite evidence of arterial extravasation from the spleen. Active extravasation from arterial branches of right hepatic lobe was noted. A microcatheter and microwire were then used to selectively cannulate the feeding segmental branches and gel-foam was injected. Repeat angiography demonstrated cessation of abnormal contrast extravasation as well as preserved flow within portions of the right hepatic segmental arteries and the left hepatic artery. Initial assessment following the procedure revealed a weak right sided femoral pulse with absent distal pulses. Ultrasound revealed occlusion of right external iliac artery secondary to thrombus. In addition, there was absent signal of the common femoral artery with reconstitution of the deep femoral and superficial femoral artery.

Due to the complexity of injuries, the patient was transferred to a higher level of care at an outside facility for continued medical management and vascular intervention. The patient was started on anticoagulation, which was discontinued after one week when ultrasound revealed resolution of the right external iliac artery clot. She also underwent pinning of the left femoral neck fracture. During her hospital course, the patient was also found to have abdominal compartment syndrome. She underwent a laparotomy where part of the terminal ileum was noted to be devascularized. This resected and the abdomen was left open. A few days later, she returned to the operating room for creation of an end ileostomy and abdominal closure. Following this procedure, the patient had acute changes to her abdominal examination, and she returned to the operating room where bile leakage from the head of the pancreas was found. Drains were placed and temporary abdominal closure was performed. Two days afterward, the patient’s abdomen was closed and a wound vac was placed. Nasogastric tube feeds were initiated 2 weeks later, followed by an oral soft diet. After successful tolerance of diet, the patient was deemed stable for discharge.

## Discussion

The usage of minimally invasive techniques for hemodynamically stable patients has become standard of care in the case of blunt liver trauma [1]. The decision between conservative versus operative management is dictated by the patient’s hemodynamic status rather than the grade of injury. If the patient is stable, the provider is able to utilize imaging modalities, such as CT of the abdomen and pelvis. If there is evidence of extravasation, angioembolization serves as a therapeutic option. Although algorithms regarding blunt liver trauma exist and can provide guidance for management, clinician judgment and discretion plays a key part in treatment [4, 5].

This case report presented an 18-month-old that suffered a traumatic grade IV liver laceration. The decision to proceed with endovascular management was based on the hemodynamic instability and presence of extravasation. Despite hemorrhage control of the liver with angioembolization, the case was complicated by arterial thrombus formation. This occlusion prompted the decision to transfer them to a facility with more resources and pediatric expertise.

The type of endovascular treatment used in the management of this patient involved gel-foam (gelatin sponge) embolization. The mechanism of gel-foam includes vessel occlusion of the vascular lumen subsequently causing thrombus formation, which, in turn, shortens coagulation time to form that thrombus. This ultimately assists in halting or temporarily seizing the site of hemorrhage [5, 6]. Complications of gel-foam are uncommon, but include non-target embolization, ischemia, and infection [7–9].

Studies around pediatric liver lacerations have revealed success with conservative therapies when compared with operative interventions [2, 3]. However, nonoperative management for traumatic liver injuries with endovascular therapies remains extremely limited for pediatric populations in the current literature. Based on our experiences highlighted in this report, training that includes angioembolization in the pediatric population is clearly warranted to make this therapy more widely available rather than isolated to specialized centers. In addition, as there is limited literature on this topic, further research should be conducted to determine the efficacy of angioembolization in blunt liver trauma in the pediatric population. Data extrapolated from these studies can then be used to formulate guidelines and algorithms that can direct trauma surgeons that face similar scenarios in the pediatric population.
